# Case Report: Whole-Exome Sequencing With MLPA Revealed Variants in Two Genes in a Patient With Combined Manifestations of Spinal Muscular Atrophy and Duchenne Muscular Dystrophy

**DOI:** 10.3389/fgene.2021.605611

**Published:** 2021-03-10

**Authors:** Yu Xia, Yijie Feng, Lu Xu, Xiaoyang Chen, Feng Gao, Shanshan Mao

**Affiliations:** ^1^National Clinical Research Center for Child Health, Department of Neurology, The Children's Hospital, Zhejiang University School of Medicine, Hangzhou, China; ^2^National Clinical Research Center for Child Health, Department of Developmental and Behavioral Pediatrics, The Children's Hospital, Zhejiang University School of Medicine, Hangzhou, China

**Keywords:** spinal muscular atrophy, Duchenne muscular dystrophy, synchronous diseases, whole-exome sequencing, MLPA, Nusinersen (Spinraza)

## Abstract

Spinal muscular atrophy (SMA) and Duchenne muscular dystrophy (DMD) are two common kinds of neuromuscular disorders sharing various similarities in clinical manifestations. SMA is an autosomal recessive genetic disorder that results from biallelic mutations of the survival motor neuron 1 gene (*SMN1*; OMIM 600354) on the 5q13 chromosome. DMD is an X-linked disorder caused by defects in the *DMD* gene (OMIM 300377) on the X chromosome. Here, for the first time, we report a case from a Chinese family who present with clinical manifestations of both two diseases, including poor motor development and progressive muscle weakness. We identified a homozygous deletion in exons 7 and 8 of the *SMN1* gene and a deletion in exon 50 of the *DMD* gene by whole-exome sequencing (WES) and multiplex ligation-dependent probe amplification (MLPA). This case expands our understanding of diagnosis for synchronous SMA and DMD and highlights the importance of various genetic testing methods, including WES, in differential diagnosis of neuromuscular diseases.

## Background

Spinal muscular atrophy (SMA) is a severe and disabling neuromuscular genetic disorder with a relatively high incidence in infants and adolescents (Lunn and Wang, 2008). The prevalence of SMA ranges from 1/6,000 to 1/10,000 in newborns (Sheng-Yuan et al., 2010). This disorder is caused by the mutations of the survival motor neuron 1 gene (*SMN1*; OMIM 600354) on the 5q13 chromosome that causes degeneration of motor neurons in the anterior horn of the spinal cord (Burghes and Beattie, 2009). The major mutations identified in SMA patients are the deletion in exon 7 or deletion in both exons 7 and 8 of the *SMN1* gene. According to the onset age and motor function differences in children with SMA, there are four subtypes of SMA: type I, type II, type III, and type IV. The other subtype (type 0) refers to the onset of SMA at birth that causes death within a few weeks (Matesanz et al., 2020). The main manifestations of SMA are progressive and symmetrical muscle weakness and atrophy in the proximal limbs and trunk, with other organs of various systems affected to varying degrees.

Duchenne muscular dystrophy (DMD; OMIM 310200) is an X-linked recessive genetic disorder caused by defects of the *DMD* gene (OMIM 300377) on the X chromosome. Although various single nucleotide variation or small indel mutations in the *DMD* gene have been reported to be associated with the risk of DMD, exonic deletions or duplications are also common. DMD affects approximately 1 in 3,600 live male babies (Fox et al., 2020). The clinical manifestations of DMD are progressive muscle weakness and motor function regression, accompanied by positive Gower sign and characteristic pseudomuscular hypertrophy and extensive muscle atrophy.

Although the genetic mechanisms of SMA and DMD are quite different, their clinical manifestations share various similarities, including progressive weakness and muscle atrophy of the proximal limbs and trunk. In addition, both diseases may lead to multiple system problems. Hence, once either one of these two diagnoses is confirmed, the other will usually be discounted from consideration, as the possibility of occurrence of both neuromuscular comorbidities is extremely small.

In this case, we report a Chinese patient presenting with combined features of SMA and DMD. Deficiencies in two related genes, the *SMN1* and the *DMD* genes, were identified by whole-exome sequencing (WES) and multiplex ligation-dependent probe amplification (MLPA). To our knowledge, patients known to carry multiple causative gene mutations are uncommon because typical “syndromes” usually direct clinicians to one specific diagnosis. Here, we share our experience in the diagnosis of such a rare case.

## Case Presentation

The 11-month-old male patient was born at full term to non-consanguineous parents with no complications. His birth weight and height were both normal. He presented with poor motor development and muscle weakness that gradually progressed after birth. He began to raise his head in prone position when he was at 43 days old, and was able to kick frequently at 3 months old, but he was still unable to grasp his feet at 6 months old. Subsequently, he had decreased muscle strength in lower limbs with diminished tendon reflexes at 7 months old. He presented at our hospital with muscle weakness and delayed development. Before coming to our hospital, he had received rehabilitation exercise treatment at the local agency.

At the initial visit when he was 6 months old, we first performed neurological and physical examinations of the patient and found decreased muscle strength, especially in the lower limbs, which could not induce the tendon reflex. The remainder of his examination was normal. Next, clinicians evaluated his developmental level based on clinical manifestations. The patient did not reach the milestone for motor development of rolling over and could not sit straight or pronate alone. However, his cognitive development was normal, and he had no significant medical history.

We reviewed the patient's family history and found that he belonged to a Chinese family with four members of two generations, including two males and two females. None of the other three family members exhibited any clinical anomalies.

Furthermore, the patient's electromyogram (EMG) showed a large number of positive sharp waves and fibrillation. High-amplitude coupled with long-duration motor unit potentials (MUPs) were also found in examined muscles, including the tibialis anterior, peroneus, rectus abdominis, and deltoid. No sensory deficits were found in nerve conduction studies. All the above findings indicated motor neuron damage.

Muscle weakness is a chronic and progressive symptom often caused by neuromuscular transmission disorders. It occurs at an early stage with specific clinical manifestations, which has a special relationship with genetic factors. The various causes of muscle weakness can be divided into neurogenic diseases and muscular diseases; therefore, genetic testing, electromyography, and laboratory inspection are necessary for diagnosis. In this case, muscle weakness, poor motor development, and spinal cord anterior horn cell injury suggested the possibility of SMA, a genetic disorder of congenital muscle weakness that was common in infancy.

## Laboratory Investigations and Test Results

To confirm the diagnosis, patient's peripheral blood samples were tested to check whether laboratory indicators were normal or abnormal. We found elevated levels of creatine kinase (CK) level (5,893 U/L) and other indicators, including alanine aminotransferase (ALT), aspartate aminotransferase (AST), and creatine kinase-MB (CK-MB) enzyme. Findings of other laboratory examinations were normal. We also tested the peripheral blood samples of the other three family members and unexpectedly found that the CK level of the patient's sister significantly increased to 6,906 U/L and that her ALT, AST, lactate dehydrogenase (LDH), and CK-MB levels were all increased as well. Neither of the parents had abnormalities in laboratory examinations. Since CK level could reflect myocardial damage, we also performed electrocardiography and echocardiography on the patient and three other family members. However, no abnormalities were found in the electrocardiograms or in the echocardiographs (Supplementary Table 1).

The clinical and laboratory evidence, including delayed motor development since birth, muscle weakness, and electromyography showing anterior horn cell pathology, indicated the neuromuscular genetic disorder called SMA. MLPA was therefore performed, which is considered the gold standard for diagnosing SMA. Thus, targeted genetic testing on peripheral blood sample from the patient was performed to make a molecular pathogenic diagnosis. SALSA MLPA probemix P021-B1 was performed, which contained 32 probes. Four probes were specific for sequences in exons 7 or 8 of either SMN1 or SMN2, while 17 probes annealed for the coexisting sequence of SMN1 and SMN2. One probe for NAIP gene, and remaining 10 probes were reference. In this technique, the 1-ml blood sample was centrifuged multiple times to extract DNA. The DNA was denatured at a high temperature, and polymerase chain reaction probes were used to hybridize with the targeted sequence. After probes was hybridized with the target sequences, two oligonucleotides would be ligated into a single probe, which was connected specifically for PCR amplification. The PCR product contains 32 amplicons of different lengths, which were separated by capillary electrophoresis. Each amplicon signal would be normalized with references and standard sample, which contains two copies of SMN1 and SMN2 genes.

**Figure 1:**
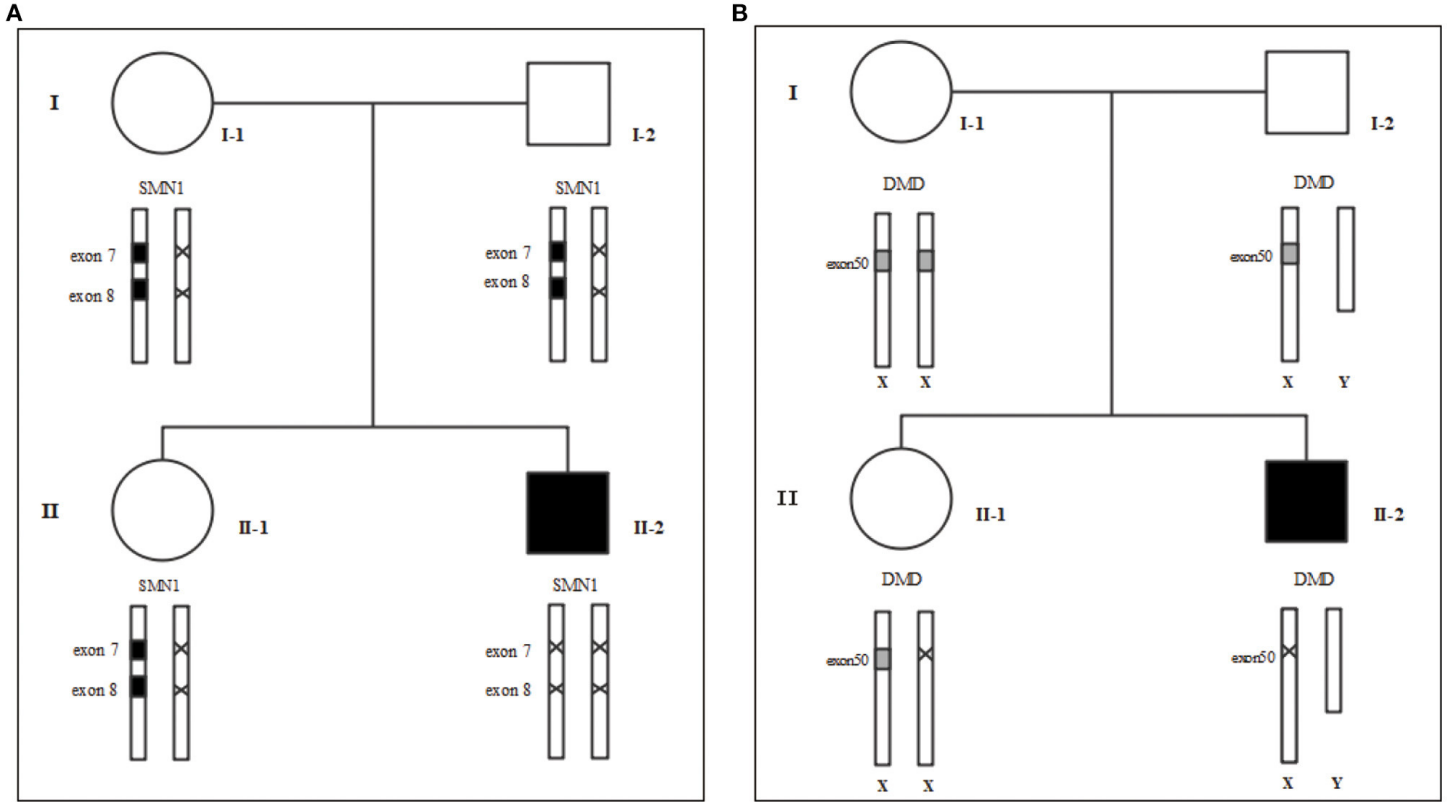
Family tree. **(A)** MLPA for SMA **(B)** MLPA for DMD. We use “×” to indicate the deletion. I-1: Mother's gene report showed only one copy in exons 7 and 8 of SMN1, and there was no deletion or duplication of the *DMD* gene; I-2: Father's gene report showed that *SMN1* in exon 7 and exon 8 had heterozygous deletions; II-1: the 2-year-old sister's gene report showed only one copy of *SMN1* in exons 7 and 8 and a heterozygous deletion in exon 50 of the *DMD* gene; II-2: The patient's gene report showed homozygous deletions of *SMN1* in exon 7 and exon 8, and a homozygous deletion in exon 50 of the *DMD* gene.

**Figure 2:**
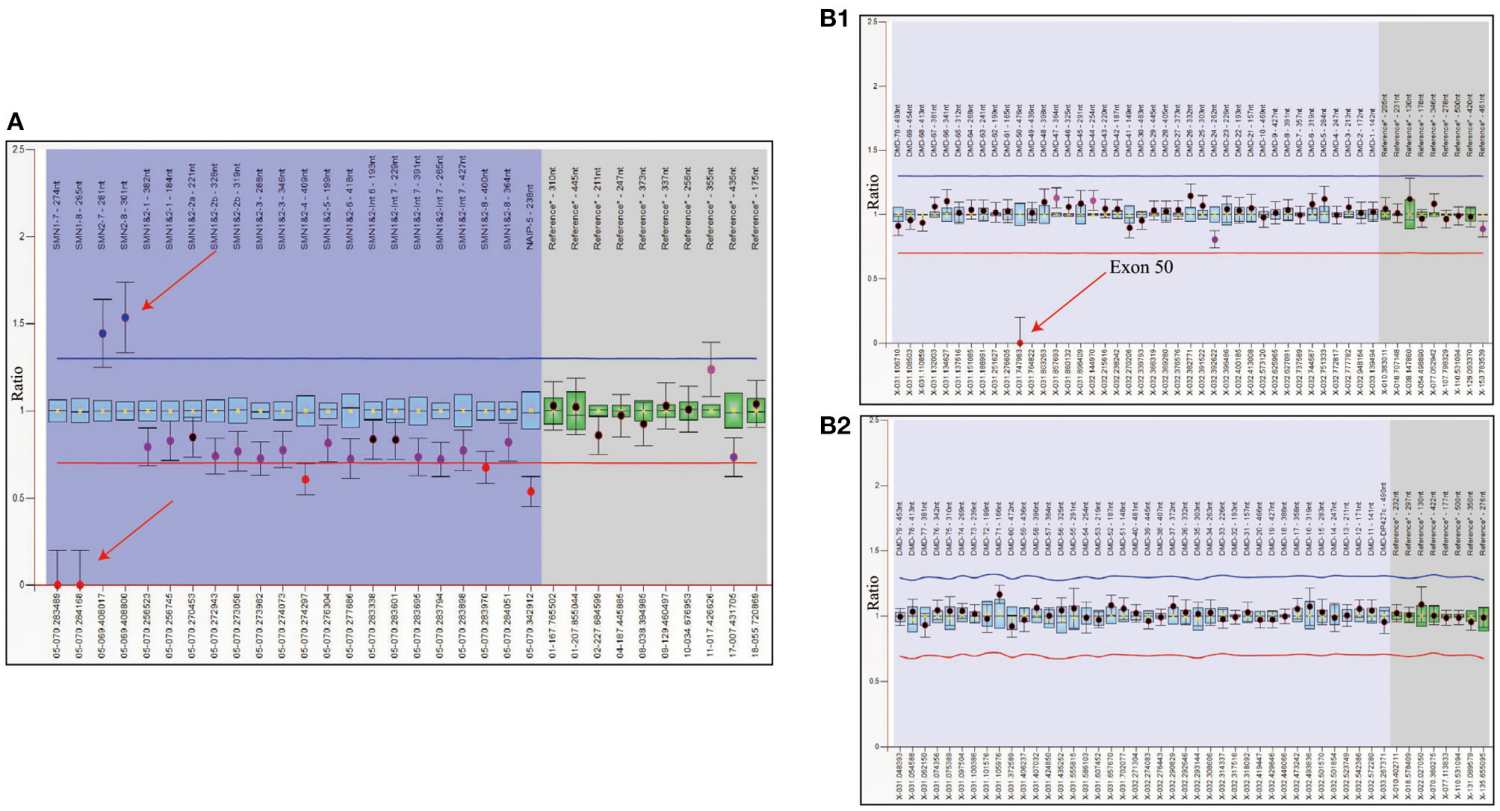
WES analysis of patient showed the complete deletion of *DMD* gene exon 50. **(A)** Integrative genomics view of patient's Xp21.1, compared with a normal reference sample. **(B)** IGV enlarged image of *DMD* gene exon 50 confirmed the mutation.

Following the standard testing procedures, MLPA for *SMN1* was performed for the first time and the results confirmed that the patient had zero copy of *SMN1* exons 7 and 8, supporting the diagnosis of SMA (Figure 2). To verify the accuracy of the test result, we also performed MLPA on blood samples from the remaining three family members. The results showed that the patient's parents and sister all carried heterozygous deletion in exons 7 and 8 of *SMN1*, indirectly confirming the patient's diagnosis of SMA (Figure 1A).

The CK levels are elevated in patients with all types of neuromuscular disorders and can be used as a primary screening marker, especially for diseases that cause myogenic damage. CK is an essential component of neonatal DMD screening due to the high sensitivity of CK for initial screening of DMD (Fox et al., 2020). Most patients with SMA may have mild to moderate CK elevation (Matesanz et al., 2020), but no more than 10 times the normal value. A diagnosis of SMA alone was insufficient to explain the elevation of CK in this patient, because his sister was an SMA carrier with increased levels of CK and CK-MB levels, but had no cardiovascular disease. Abnormal CK levels in siblings prompted us to reconsider the previous judgments about the patient's diagnosis and indicated that DMD or other neuromuscular disorders with similar clinical presentation may also need to be taken into account.

**Figure 3:**
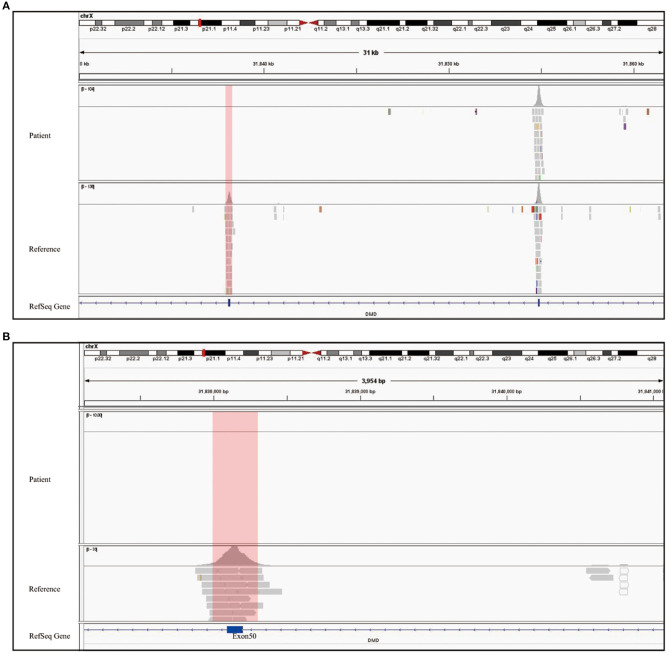
Genetic test report of SMA and DMD. **(A)** MLPA report shows 0 copy of exons 7 and 8 of *SMN1*, 3 copies of exons 7, and 8 of *SMN2*. The left arrow refers to *SMN1* exons 7 and 8, and the right one refers to exons 7 and 8 of *SMN2*. The blue region represents the *SMN1* probe, *SMN2* probe, and SMN1+2 probe shown on the top of the diagram. The gray region shows the reference probe on different chromosome. All data were normalized by the reference and standard, which contains two copies of *SMN1* and *SMN2*. Therefore, the *Y* axis is the ratio compared to standard's copy number. **(B)** The two report together presented a whole exon detection of *DMD* gene, and B1 only showed homozygous deletion in exon 50 while other exons are one copy. There is no copy number variant shown in B2. The blue region refers to exons 1–79 of the *DMD gene*, and the gray one refers to the reference probe for normalization.

To rule out other neuromuscular disorders and to verify the inference of DMD, WES was subsequently performed on all neuromuscular disorders to obtain a comprehensive differential diagnosis. WES is another popular genetic test that can cover the coding sequence of most human genes and over 99% of disease-related gene regions by measuring exons, which only account for 2% of the genome sequence. The library was prepared by shearing 1 mg of genomic DNA into small fragments of 200–300 bps. The methods used for target capture, enrichment, and elution followed protocols of techniques such as MLPA with slight modifications (van Dijk et al., 2018). The results of WES also confirmed the deletion of *SMN1* exons 7 and 8. In addition, the result also revealed a deletion of exon 50 and some intron regions of the *DMD* gene, in which it may contribute to the occurrence of DMD (Figure 2). The hemizygous mutation c.1699C>T found in the ABCD1 gene of this patient was likely to be pathogenic. However, he is only a 1-year-old boy and there were no abnormalities related to X-linked adrenoleukodystrophy (X-ALD), the abnormal motor development of the young patient would not be caused by X- ALD by now. A long-term follow-up is very necessary for this patient to observe the future process of this mutation. To verify this finding, we immediately applied MLPA for the *DMD* gene in this patient, and the results showed a hemizygous deletion of exon 50 of the *DMD* gene, directly confirming the diagnosis of DMD (Figure 3). Moreover, we also performed MLPA on the *DMD* gene of three other family members and found that the sister had a heterozygous deletion of the *DMD* gene exon 50, while the parents did not have any deletion or duplication in this gene (Figure 1B).

Finally, after this series of tests, we confirmed that the boy had synchronous SMA and DMD and that his sister was a dual carrier of both SMA and DMD.

## Discussion and Concluding Remarks

### Phenotype and Mechanism

In this case study, we found variants of the *SMN1* and *DMD* gene in a single family. The patient presented with combined features of both SMA and DMD due to deletion of exon 7 of *SMN1* and exon 51 of the *DMD* gene, respectively, while the other members all showed normal clinical manifestations (Figure 1).

Considering that SMA and DMD are differential diagnoses to each other, the diagnosis in our patient might not have been achieved for a relatively long period due to the lack of visual characteristics of DMD. Furthermore, EMG results only indicated moderate CK increase and neurogenic damage, so the diagnosis of DMD might be missed if the standard diagnostic process had not been strictly followed. Patients with synchronous SMA and DMD are quite rare among all cases of genetic disease due to the low prevalence of both diseases in the population. After a literature review, we found only one family with both SMA and DMD, which was reported many years ago by a team from the University of Gothenburg. In that case, the couple had seven pregnancies and had aborted five fetuses, including one boy who was diagnosed with DMD, two DMD carriers, and one with a combination of DMD and type I. Following the doctor's advice, one fetus highly suspected of having SMA and carrying DMD was eventually aborted (Oldfors et al., 1994). Therefore, our patient is the first case in China and the only reported living case in the world.

There have also been reports of two different inherited neuromuscular disorders in the same family or in the same patient, as listed in Table 1 (Afifi et al., 1965; Pongratz et al., 1976; Shapira et al., 1981; Matsumura et al., 1991; Oldfors et al., 1994; Casado et al., 1995; Dubrovsky et al., 1995; Monnier et al., 2000; Tojo et al., 2000; Bernard et al., 2017), suggesting the possibilities of different rare diseases of similar types occurring simultaneously. When the results of a particular test cannot be entirely explained by one single disease, it is necessary to consider the existence of another similar disease. For example, subtle differences between different muscle bundles observed in muscle biopsy can lead to different diseases. Considering that two or more diseases may occur in the same patient, clinicians should carefully observe the clinical manifestations of patients, pay attention to each test result, complete the differential diagnosis, and, most importantly, conduct a complete genetic test. If necessary, the combined application of multiple testing methods with different advantages can be used for diagnosis, as in our case. Only in this way can we avoid missing the potential diagnosis with doubt.

**Table 1:** Cases with co-occurrence of two genetic diseases in various families.

	**Disease 1**	**Disease 2**	**Gene 1**	**Gene 2**	**Family history**
1. Oldfors et al. (1994)	DMD	SMA	DMD (XR)	SMN (AR)	① A boy with DMD and SMA ② A boy with DMD ③ Two girls carried DMD ④ A fetus with SMA and carried DMD (terminate pregnancy;)
2. Matsumura et al. (1991)	FCMD	DMD	FKTN (AR)	DMD (XR)	① A boy with DMD ② Three boys with FCMD
3. Matsumura et al. (1991)	MD	DMD	DMPK (AD)	DMD (XR)	① A boy with MD and DMD ② One of his two sisters, his mother, three maternal uncles, and maternal grandfather all had MD
4. Monnier et al. (2000)	CCD	NM	RyR1 (AD)	TPM3 and ACTA1 (AD)	① II:8 A woman with CCD and TPM3 gene recombination ② III:1 III:2 Two men with CCD and TPM3 gene recombination ③ IV:1 IV:2 Two boys with CCD and TPM3 gene recombination
5. Tojo et al. (2000)	CCD	CNMDU1	RyR1 (AD)	Related to RyR1 (AD)	① A man with CCD ② His son with CNMDU1 or synchronous with CCD and CNMDU1
6. Afifi et al. (1965)	CCD	NM	RyR1 (AD)	TPM3 and ACTA1 (AD)	① A woman with CCD and NM ② Her daughter with CCD
7. Casado et al. (1995)	CCD	NM	RyR1 (AD)	TPM3 and ACTA1 (AD)	① A woman with CCD and NM ② Two of her sons with NM
8. Pongratz et al. (1976)	CCD	CNMDU1	RyR1 (AD)	Related to RyR1 (AD)	① A woman suffered with CCD ② Two of her daughters with CNMDU1
9. Bernard et al. (2017)	ALS	HD	Not clear	HTT (AD)	① A man with ALS and duplication in HTT gene ② His mother suffered with HD
10. Shapira et al. (1981)	NM	MM	TPM3 and ACTA1 (AD)	mtDNA (maternal inheritance)	① A boy with NM ② A boy with NM ③ Their father with MM

*AR, Autosomal recessive; AD, Autosomal dominant; XR, X-linked recessive inheritance; DMD, Duchenne Muscular Dystrophy; SMA, Spinal Muscular Atrophy; FCMD, Fukuyama-type congenital muscular dystrophy; MD, Myotonic dystrophy; CCD, Central core disease; NM, Nemaline myopathy; FKTN, Fukutin gene; DMPK, Dystrophia myotonica protein kinase gene; RyR1, Ryanodine receptor gene; TPM3, α-tropomyosin; ACTA1, α-actin*.

In our case, there remains another outstanding question. How can the patient's mother, as an SMA carrier with no deletion or mutation of the *DMD* gene exon 50, given birth to a DMD patient whose sister is also a DMD carrier, and both of them carry abnormalities in exon 50 of the *DMD* gene? With repeated tests for DMD integrity in her peripheral blood, throat swabs and urine, we found no deletion or duplication of exon 50 of the *DMD* gene in these cells. Thus, it is speculated that she has a high possibility of having a germline mosaic mutation. Mosaicism is defined as the presence of cells with at least two different genotypes in an individual (Helderman-van den Enden et al., 2009). Germline mosaicism may originate from mutations occurring during sperm or egg formation that affect multiple germ cells, while somatic cells in the parent are not affected (Biesecker and Spinner, 2013).

We know that the risk of recurrent DMD due to germline mosaicism in non-carrier women is estimated at 14–20% (Helderman-van den Enden et al., 2009). In this family, in the absence of medical intervention, the recurrence rate of SMA in infants was 25% and the recurrence rate of DMD in boys was 14–20%. The co-occurrence of SMA and DMD is also much higher than that of the general population. Therefore, if the woman wants to have another child, we would advise her to choose *in vitro* fertilization and prenatal genetic diagnosis to ensure that the fertilized embryo would not carry such genetic mutations or deletions. However, giving the possibility of false-positive results of prenatal examinations, further complications can still occur, as an unknown degree of mosaicism may result in the termination of a fetus with a normal phenotype.

The patient's sister had one copy of *SMN1*, two copies of *SMN2*, and heterozygous deletion of the *DMD* gene exon 50, suggesting that she was a dual carrier of SMA and DMD. Furthermore, her CK level was 6,906 U/L, combined with abnormal elevations of other liver function indicators. In general, SMA carriers do not have any signs of motor dysfunction, while up to 20% of female DMD carriers have mild to moderate muscle weakness, and an elevation of CK level can be observed in 50–60% of carriers. In addition, 8% of female carriers have dilated cardiomyopathy (Fox et al., 2020). These may be related to autosomal transposition of the Xp21 in DMD carriers, which has been demonstrated in several other studies. Though no abnormality was observed in the electrocardiogram and ultrasonograph of the sister, long-term follow-up for the cardiovascular system is needed. If she becomes pregnant, a comprehensive screening of the fetus for prenatal neuromuscular disorders is necessary.

### Current Treatment and Management Status

In the past decade, there has been increasing attention on the treatment of genetic diseases (Dolgin, 2019; Doudna, 2020). It is widely accepted that significant breakthroughs have been made for the treatment of SMA and DMD. SMA drugs are mainly divided into two categories, SMN-dependent and SMN-independent (Smith, 2020). At present, three SMN-dependent drugs have been approved for marketing, namely, the SMN1-replacement drug Zolgensma (Mendell et al., 2017), the SMN2-targeted drug Nusinersen (Mercuri et al., 2018), and Risdiplam (Yu et al., 2020). Each of these has achieved significant results after marketing authorization. Nevertheless, Zolgensma is currently indicated for the treatment of patients who have SMA type I or up to 3 copies of the SMN2 gene, and the most crucial issue is whether the safety of high-dose virus vector and systemic administration requires further evaluation (Hoy, 2019). Because Zolgensma has not been approved for marketing in China, the patient started treatment with Nusinersen, which is an accessible and safe therapy for him, and he has achieved improvements in several aspects. As a genetic modifier, Nusinersen does not completely restore SMN protein levels in all body tissues (Singh, 2019). Thus, the development of permanent and effective strategies based on correcting endogenous genetic mutations may offer the possibility of a thorough cure for this lethal neuromuscular disorder.

The therapeutic management of DMD includes glucocorticoid, gene-addition, exon-skipping, stop codon read through, genome-editing, and many other types of therapy (Grages et al., 2020). Drugs already on the market include the glucocorticoid drug Deflazacort, the exon-skipping drugs Eteplirsen and Golodirsen, and the stop codon read through drug Ataluren (Korinthenberg, 2019). Considering the unexplainable complication such as related myositis, long-term corticosteroid therapy can partially alleviate the pathological phenotype caused by DMD, but cannot fundamentally restore dystrophin expression. Other therapies include exon-skipping drugs, stop codon read through drugs, and gene-addition, and more clinical trial evidence is needed to prove their effectiveness. Gene editing therapy is still in the preclinical research stage (Fernández-Ruiz, 2020). Precision therapy for DMD has not yet been approved for use in China and even no clinical trial has been launched. In conclusion, treatment options for DMD in China are limited and need to be carefully selected.

After the patient was diagnosed, we explained to the patient's parents the nature of the disease, the difficulties faced in the treatment of the disease, the currently available drug treatment, and the therapeutic situation in China, and described in detail the possible side effects and burden. The parents received a thorough understanding of the disease and accepted treatments of SMA. Because of the inaccessibility of therapeutic drugs and concerns over the side effects of glucocorticoid, the parents chose not to utilize drug to treat DMD, but instead a joint intervention by a multi-disciplinary team consisting of clinicians, caregivers, gene therapy experts, and other specialties to help delay the process of DMD. We will follow up with him, timely evaluate the declined level of his lung function, joint contracture, scoliosis, and other changes, and immediately carry out symptomatic treatment.

After an initial treatment plan was formulated, the patient began to receive Nusinersen treatment immediately. After admission, he underwent a detailed evaluation including routine blood, blood coagulation function, biochemical, routine urine, myocardial marker, cardiac ultrasound, and detailed motor function assessments. To date, he has completed five doses since he started to receive Nusinersen injections. Because drug therapy in combination with family rehabilitation started at the age of 6 months, the patient's motor function improved substantially according to his parent's feedback.

Two motor function assessments were made before the fourth (day 63) and fifth (day 185) doses of Nusinersen. Compared with day 0 (before the first injection), the Children's Hospital of Philadelphia Infant Test of Neuromuscular Disorders (CHOP INTEND) score increased from 43 to 45 and 46 points, respectively, and the Hammersmith Infant Neurological Exam (HINE-2) score increased from 6 to 10 and 13 points, respectively. Apart from these observations, his parents also reported that after the third injection, he was able to roll back and forth continuously twice at a time without assistance. He could control his head movements in the prone position and could prop up his upper body with his upper limbs away from the plane. Moreover, he was able to sit more steadily and had increased strength of his lower limbs. At present, the patient's condition is relatively stable, with no further progress, and improved exercise ability. His parents were pleased with the improvement in their son and would like to continue the injection treatment to see if there would be a greater motor improvement in the future (Supplementary Figure 1).

The two different genetic testing methods have their own advantages and shortcomings. MLPA is easy to operate, has short reporting time, and is particularly sensitive to single-gene deletions or duplications. However, it cannot detect point mutations, minor deletions or duplications (especially those not reported before), or balanced translocation of chromosomes. In addition, MLPA has stringent requirements for measuring concentration and sample quality. By contrast, WES is more suitable for high-throughput and large-scale gene sequencing, which can more comprehensively detect point mutations and copy number variations in genes associated with genetic disease. When clinicians are unable to identify potential targeted gene, WES can obtain inspection data with larger coverage at one time, which is currently a better choice than MLPA. At present, however, MLPA is still the gold standard in China, and WES is used to assist in detecting more complicated situations. Thus, since we only suspected other neuromuscular diseases and could not directly confirm their identity, WES was a better method to provide differential diagnosis in this case. MLPA can validate the results and help to make a comprehensive diagnosis.

The difficulty and challenge in the diagnosis of this case is the combination of two diseases with extremely low probability of co-occurrence and similar clinical manifestations and laboratory examination characteristics, which usually require differential diagnosis. A disease with mild symptoms can easily be missed if the more apparent disease always covers its manifestations. In our case, it was the abnormality in CK level that attracted the clinicians' attention and prompted us to further explore the cause.

In conclusion, we here report a rare case of synchronous SMA and DMD. SMA and DMD are no longer incurable disease, as several therapeutic drugs are now available for both conditions, although the effects of DMD still need further investigation. Considering the treatment dilemmas for such a unique patient, a thorough mastery of the pathogenesis and clinical manifestations of both rare diseases is urgently required. To date, we still lack targeted, standardized treatment options for similar patients. We recommend tailored targeted therapies and multi-disciplinary management to adapt to the clinical symptoms of each child at different stages. Furthermore, the diagnosis of rare genetic diseases requires careful examination and genetic testing by clinicians. Especially in this case, the combined application of multiple methods should be adopted as soon as possible to make a definitive and comprehensive diagnosis. Precise therapy is not far away from us.

## Data Availability Statement

The original contributions presented in the study are included in the article/Supplementary Material, further inquiries can be directed to the corresponding author/s.

## Ethics Statement

The studies involving human participants were reviewed and approved by Children's Hospital of Zhejiang University School of Medicine Ethics Committee. Written informed consent to participate in this study was provided by the participants' legal guardian/next of kin. Written informed consent was obtained from the individual(s), and minor(s)' legal guardian/next of kin, for the publication of any potentially identifiable images or data included in this article.

## Author Contributions

YX designed study, collected and analyzed the data, and drafted the manuscript. YF collected and analyzed the data and drafted the manuscript. LX provided patient care and treatment. XC provided patient care and assessment for motor function. FG provided patient care and neurology expertise. SM designed study, collected and analyzed the data, revised the manuscript, supervised the study, and provided final approval. All authors contributed to the article and approved the submitted version.

## Conflict of Interest

The authors declare that the research was conducted in the absence of any commercial or financial relationships that could be construed as a potential conflict of interest.
