# Corrigendum: Case Report: Whole-Exome Sequencing With MLPA Revealed Variants in Two Genes in a Patient With Combined Manifestations of Spinal Muscular Atrophy and Duchenne Muscular Dystrophy

**DOI:** 10.3389/fgene.2021.684042

**Published:** 2021-06-01

**Authors:** Yu Xia, Yijie Feng, Lu Xu, Xiaoyang Chen, Feng Gao, Shanshan Mao

**Affiliations:** ^1^National Clinical Research Center for Child Health, Department of Neurology, The Children's Hospital, Zhejiang University School of Medicine, Hangzhou, China; ^2^National Clinical Research Center for Child Health, Department of Developmental and Behavioral Pediatrics, The Children's Hospital, Zhejiang University School of Medicine, Hangzhou, China

**Keywords:** spinal muscular atrophy, Duchenne muscular dystrophy, synchronous diseases, whole-exome sequencing, MLPA, Nusinersen (Spinraza)

In the original article, there was an incorrect information. “The indications for Zolgensma are currently confined to the treatment of type I” is inaccurate. A correction has been made to the Section: Discussion and Concluding Remarks, Subsection: Current Treatment and Management Status, Paragraph 1: In the past decade, there has been increasing attention on the treatment of genetic diseases (Dolgin, 2019; Doudna, 2020). It is widely accepted that significant breakthroughs have been made for the treatment of SMA and DMD. SMA drugs are mainly divided into two categories, SMN-dependent and SMN-independent (Smith, 2020). At present, three SMN-dependent drugs have been approved for marketing, namely, the SMN1-replacement drug Zolgensma (Mendell et al., 2017), the SMN2-targeted drug Nusinersen (Mercuri et al., 2018), and Risdiplam (Yu et al., 2020). Each of these has achieved significant results after marketing authorization. Nevertheless, Zolgensma is currently indicated for the treatment of patients who have SMA type I or up to 3 copies of the SMN2 gene, and the most crucial issue is whether the safety of high-dose virus vector and systemic administration requires further evaluation (Hoy, 2019). Because Zolgensma has not been approved for marketing in China, the patient started treatment with Nusinersen, which is an accessible and safe therapy for him, and he has achieved improvements in several aspects. As a genetic modifier, Nusinersen does not completely restore SMN protein levels in all body tissues (Singh, 2019). Thus, the development of permanent and effective strategies based on correcting endogenous genetic mutations may offer the possibility of a thorough cure for this lethal neuromuscular disorder.

The authors apologize for this error and state that this does not change the scientific conclusions of the article in any way. The original article has been updated.
