# Supplementary material for: Case Report: Whole-Exome Sequencing With MLPA Revealed Variants in Two Genes in a Patient With Combined Manifestations of Spinal Muscular Atrophy and Duchenne Muscular Dystrophy
Source: Front Genet. 2021 Mar 10;12:605611. doi: 10.3389/fgene.2021.605611 (PMC7987946; doi:10.3389/fgene.2021.605611)
Supplement: Supplementary file 2 [file Table_1.DOC]

|  | **patient** | **sister** | **mother** | **father** |
| --- | --- | --- | --- | --- |
| creatine kinase (CK) U/L | 5893 | 6906 | 217 | 189 |
| alanine aminotransferase (ALT) U/L | 118 | 233 | 16 | 37 |
| Aspartate aminotransferase (AST) U/L | 213 | 163 | 24 | 51 |
| creatine kinase-MB (CK-MB) U/L | 413 | 165 | 29 | 36 |
| lactate dehydrogenase (LDH) U/L | 1517 | 556 | 246 | 190 |
| Electrocardiograms | Normal | Normal | Normal | Normal |
| Echocardiographs | Normal | Normal | Normal | Normal |
| head MRI | Normal | - | - | - |
| electromyogram (EMG) | Neurogenic damage | - | - | - |
